# The 2026 Nucleic Acids Research database issue and the online molecular biology database collection

**DOI:** 10.1093/nar/gkaf1427

**Published:** 2025-12-23

**Authors:** Daniel J Rigden, Xosé M Fernández

**Affiliations:** Department of Biochemistry, Cell and Systems Biology, Institute of Systems, Molecular and Integrative Biology, University of Liverpool, Crown Street, Liverpool L69 7ZB, United Kingdom; IQVIA Ltd., The Point, 37 North Wharf Road, London W2 1AF, United Kingdom

## Abstract

The 2026 Nucleic Acids Research database issue has 182 papers from across biology and neighbouring fields. Eighty-four of these papers describe new databases, while 86 are updates on databases that have previously appeared here. Twelve more papers cover databases most recently published elsewhere. New nucleic acid databases include NapRNAdb for noncapped RNA and GlycoRNAdb. Protein structure is covered by updates from wwPDB members and the AlphaFold Database; SMART, PROSITE, and eggNOG cover domains and families. The Open Enzyme Database and QSproteome are new community-orientated initiatives. JoGo covers hierarchically named and contextualised human haplotypes in the issue’s first Breakthrough paper; So3D provides genuinely 3D spatial transcriptomics in the other. Foundational databases Genenames.org and Gene Ontology also provide updates. The Database Issue is freely available on the Nucleic Acids Research website (https://academic.oup.com/nar). At the NAR online Molecular Biology Database Collection (http://www.oxfordjournals.org/nar/database/c/), over the past year, 899 entries were reviewed, 96 new resources added, and 319 discontinued URLs removed, bringing the total number of databases to 2173.

## New and updated databases

The 33rd Nucleic Acids Research database issue contains 182 papers spanning biology and related areas. The issue features 84 papers describing new databases (Table 1) as well as 12 on databases most recently published elsewhere (Table 2). There are also 86 papers on resources that have previously appeared here. As usual, the issue leads with updates from the major database providers at the European Bioinformatics Institute (EBI), the U.S. National Center for Biotechnology Information (NCBI), and the National Genomics Data Center (NGDC) in China [1–3]. Notably, the last of these now includes the aptly named BIG search, which scans across databases not just of the NGDC and partners, but also from the EBI and NCBI. The usual categorization of remaining papers then follows: (i) nucleic acid sequence and structure, transcriptional regulation; (ii) protein sequence and structure, proteomics; (iii) metabolic and signalling pathways, enzymes and networks; (iv) genomics of viruses, bacteria, protozoa, and fungi; (v) genomics of human and model organisms plus comparative genomics; (vi) human genomic variation, diseases, and drugs; (vii) plants; and (viii) other topics. As always, many databases span multiple categories, so readers are encouraged to browse the whole issue.

**Table 1.** Descriptions of new databases in the 2026 NAR database issue

Database Name	URL	Short description
AMRnet	https://www.amrnet.org/	Genome-derived AMR surveillance data
ANPDB	https://african-compounds.org	African Natural Products Database
ARKbase	https://datascience.imtech.res.in/anshu/arkbase/	Antimicrobial Resistance Knowledgebase
ASTRA	https://astra-db.com/	The Atlas of STress Response Activity
CCCdb	http://www.licpathway.net/cccdb/index.php	Cell-cell communication in human and mouse
CellSNVReg	http://bio-bigdata.hrbmu.edu.cn/CellSNVReg/index.jsp	SNV-mediated regulatory perturbations in single-cell and spatial omics
ChickenGTEx Atlas	http://chicken.farmgtex.org	Chicken genotypes and phenotypes
ChromPolymerDB	https://chrompolymerdb.bme.uic.edu/	Single-Cell 3D Chromatin Structures
CircTarget	https://circtarget.cn	circRNA-target RNA interactions
ClinMAVE	https://ngdc.cncb.ac.cn/clinmave/	Clinical application of MAVE assays
CNAScope	https://cna.fengslab.com/	Pan-Cancer Copy Number Variation Database
CRESTA	https://cresta.renlab.cn/	Cellular response to external stressors transcriptome atlas
dbCAN-HGM	https://pro.unl.edu/dbCAN_HGM	CAZymes and gene clusters in human gut microbiomes
DeepSpaceDB	https://www.deepspacedb.com	Curated and interactive database of spatial transcriptomics
DisCP-Atlas	http://www.discpatlas.net	GWAS and single cell data link cellular processes to disease [CHECK]
DOO	https://DeepOceanOmics.org	Deep Ocean Omics
DynaRepo	https://dynarepo.inria.fr/	Macromolecular conformational dynamics
enhancer3D	https://3dgnome.mini.pw.edu.pl/enhancer3D/	Chromatin 3D Structure Database for Archaic and Modern Humans
ENSURE	https://trna.lumoxuan.cn/	Encyclopedia of Suppressor tRNA
EnzEngDB	http://enzengdb.org/	Enzyme Engineering Database
GEAR	https://scientist.xsrv.jp/	Diurnal and circadian gene expression in Arabidopsis
GENEasso	https://www.geneasso.net	Disease-Gene Associations GWAS by diverse methods
GeneFamily	https://gh.deepomics.org/	Mammalian gene family database
gutMSNP	https://bio-computing.hrbmu.edu.cn/gutMSNP/home	SNPs in the human gut microbiome
GlycoRNAdb	http://www.glycornadb.com	GlycoRNA
HapScoreDB	https://bcglab.cibio.unitn.it/hapscoredb	Haplotype-resolved protein-coding sequences and pLM fitness scores
HLRMDB	http://www.inbirg.com/hlrmdb/	Human long-read metagenomic database
Human-scATAC-Corpus	https://health.tsinghua.edu.cn/human-scatac-corpus/	Human scATAC-seq data
iFish	https://gonglab.hzau.edu.cn/iFish	Fish multi-omics
IGVF Catalog	https://api.catalog.igvf.org/	Impact of Genomic Variation on Function Catalog
Iridovirus Comprehensive Database	https://www.iridovirus.com/	Iridovirus sequences, epidemiology and disease management
JoGo	https://jogo.csml.org	Haplotype annotation for canonical human protein-coding genes
KnockRBP	https://knockrbp.xu-bioinfo.com	Multi-omics of RNA-binding protein knockout or knockdown
m6AConquer	http://rnamd.org/m6aconquer	Consistent Quantification of m6A RNA Modification Data
MacrocycleDB	https://macro-db.dpbio.tech/ and https://macro-db.cn/	Macrocycle compounds
MeDIC	https://medic.renci.org/	Medicines, Diseases, and Indications
MediaAssist	https://mediaassist.ncl.res.in	Serum-free cell culture media information
Meta-VR	https://www.meta-virome.org/	Metagenome-derived viruses
MetabFlow	https://bddg.hznu.edu.cn/metabflow/	Natural product metabolism
metagRoot	https://pavlopoulos-lab.org/metagroot/ or https://www.metagroot.org	Protein Families Associated with Plant Root Microbiomes
Metalog	https://metalog.embl.de/	Manually annotated metadata for metagenomic data
MetaTraits	https://metatraits.embl.de	Microbial phenotypic traits
MGDB	http://mgdb.idruglab.cn/	Molecular Glues
MGTbind	https://mgtbind.pkumdl.cn	Molecular Glue and Ternary binding Database
MicroAgroBiome	https://agrobiom.matmor.unam.mx	Metagenome-assembled genomes from crop-associated environments
MicrobialScope	https://microbial.deepomics.org/	Richly annotated genomes from bacteria, archaea, fungi, and viruses
MITE	https://mite.bioinformatics.nl/	Minimum Information about a Tailoring Enzyme Database
MolGlueDB	https://www.molgluedb.com	Molecular Glues
MouseOmics	http://www.varnatech.cn/MouseOmics	Mouse multi-omics
NapRNAdb	https://bioinformaticsscience.cn/naprnadb	Noncapped RNAs (napRNAs)
Ocean-M	https://om.biocloud.net	Multi-omics database of marine microbiomes
OED	https://openenzymedb.platform.moleculemaker.org/	Open Enzyme Database
PBMCpedia	https://web.ccb.uni-saarland.de/pbmcpedia/	Human peripheral blood mononuclear cell transcriptomics
pcsRNADB	http://pcsrnadb.cloudna.cn/#/Home	pan-cancer small RNA expression
PersADE	https://47.88.56.212/PersADE/	Personalised adverse drug events
PMADS	https://pmads-db.org	PTM–drug–disease relationships
PRIME	https://primedb.sjtu.edu.cn	Phenotypic Reference for Integrated Microbiome Enrichment
ProteoNexus	https://www.proteonexus.com/	The plasma proteome mediating complex disease
QSproteome	https://QSProteome.org	Computationally predicted oligomeric protein complexes
RaCE	https://biospace.shinyapps.io/race/	Rare Cancer Explorer
RadioPharm	https://idrblab.org/radiopharm/	Radiopharmaceuticals
ResMicroDb	https://resmicrodb.cncb.ac.cn	The respiratory microbiome in health and disease
Ribocentre-aptamers	https://aptamer.ribocentre.org/	RNA aptamers
RMpore	https://rmpore.renlab.cn/	Single-molecule RNA modification profiling via Nanopore sequencing
RNApaceDB	http://43.163.194.16/RNAPaceDB/	RNA velocities
scBrainScope	http://www.brainscopes.org or http://8.142.154.29/scBrainScope/	Cross-species multi-dimensional integrated brain atlas
scCT-DB	http://scctdb.ncpsb.org.cn	Cancer patient-derived paired pre- and post-treatment single-cell transcriptomes
scGeneBank	http://8.142.154.29/scGeneBank or http://www.genebank.info	Cross-species screening of functional gene sets at single-cell resolution
scMOVIR	https://pgx.zju.edu.cn/scmovir	Single-cell multi-omics database for viral infections and immune responses
scVMAP	https://bio.liclab.net/scvmap/	Single-cell chromatin accessibility data and causal variants
SDMap	http://bio-bigdata.hrbmu.edu.cn/SDMap/	Spatial Drug perturbation Map
SeekPCMdb	https://ngdc.cncb.ac.cn/seekpcmdb/	Protein-coding mutations in human diseases
smallBARNA	https://web.ccb.unisaarland.de/smallbarna/	Curated kingdom-wide bacterial sRNA
So3D	http://bio-bigdata.hrbmu.edu.cn/So3D or https://so3d.bio-database.com/	3D spatial omics
Spatial2GWAS	http://www.spatial2gwas.cn	Spatial transcriptomic regions linked to GWAS traits
Spatial GWAS Atlas	https://zhaolab.cpl.ac.cn/spatialgwas	Spatial transcriptomic regions linked to GWAS traits
SVAtlas	https://www.svatlas.org/	Single extracellular vesicle omics resource
TE-SCALE	https://ngdc.cncb.ac.cn/te-scale/	Transposable Element Single-Cell Analysis Landscape
TEDD	https://ngdc.cncb.ac.cn/tedd	Translation Efficiency Dynamics Database
theRNA	https://therna.renlab.cn/	Therapeutic RNA
TPDdb	https://idrblab.org/TPDdb/	Targeted Protein Degraders
TTDB	https://sysbio.gzzoc.com/ttdb/index.html	Transcriptome Turnover Database
VIRE	https://vire.embl.de	Viral Integrated Resource across Ecosystems
VirJenDB	https://www.virjendb.org	Virus genomics

**Table 2.** Updated descriptions of databases most recently published elsewhere

Database Name	URL	Short description
3D Genome Browser	https://3dgenome.fsm.northwestern.edu	3D Genome Architecture
CD-CODE	https://new.cd-code.org	Biomolecular condensates and their constituents
CIRCpedia	https://bits.fudan.edu.cn/circpediav3	Circular RNAs across 20 species
CisBP-RNA	https://www.cisbp.org/rna/	Eukaryotic RNA Binding Protein motifs
connectomeDB	https://connectomedb.org	Human ligand–receptor interactions
groov^DB^	https://groov.bio	Prokaryotic transcription factor biosensors and their properties
Massbank	https://massbank.jp/	Mass Spectral Database
OmniPath	https://omnipathdb.org/	Integrated molecular interactions, pathways, and biological annotations
PaxDb	https://www.pax-db.org/	Protein abundance at organism and tissue levels
PRECOG and related resources	https://precog.stanford.edu	Gene-outcome associations across cancer
RegNetwork	http://www.zpliulab.cn/RegNetwork/home	Gene Regulatory Networks in human and mouse
riboCIRC	http://www.ribocirc.com	Translatable circRNAs

In the ‘Nucleic acid databases’ section a number of interesting classes of RNA earn new bespoke databases: GlycoRNAdb [4] captures the expression, composition, and glycosylation sites of the recently discovered cell surface glycoRNA molecules, while aptamer structures and their diverse ligands are the focus of Ribocentre-aptamer [5]. Elsewhere, noncapped RNAs are catered for by NapRNAdb [6], which encompasses sequences, evolution, and expression of eight classes across 40 species; and bacterial sRNAs are covered by SmallBARNA [7], the content of which derives exclusively from 1000 carefully curated papers. Suppressor tRNAs, their natural occurrence and therapeutic potential, are the focus of the new ENSURE database [8]. It offers an AI-driven user query system, as many resources have introduced, and notably uses the latest AlphaFold 3 [9] to model RNA molecules. Circular RNAs are comprehensively covered by the new CircTarget [10], which captures RNA–RNA interactome data from several cross-linking methods and provides a rich context highlighting potential links to disease. This joins the returning CircleBase [11], which expands 12-fold and now covers mouse data for cross-species comparison, and two databases that are new to the issue: CIRCpedia [12], which adopts community recommendations on nomenclature and covers 20 species; and riboCIRC [13], for translatable circRNAs, which doubles in size, introduces new layers of evidence translation, and offers more comprehensive characterization of the encoded peptides. Also welcomed to the issue is CisBP-RNA [14], the popular resource for eukaryotic RNA-binding proteins and their motifs. Their update provides a helpful pipeline to assess the RNA-binding protein complement of a newly sequenced genome. Popular returning databases include RNAcentral [15], which reports two major developments—two AI tools to link literature to entries and provide summaries; and a shift in representation from sequences to genes and their transcripts. Elsewhere, NONCODE [16], the resource for long non-coding RNAs, adds transcriptomics data from millions of human single cells, and MODOMICS [17] celebrates 20 years by vastly expanding its RNA sequence content through curation of data from the Sci-ModoM database [18]. Finally, the hugely influential database of rRNA SILVA reappears [19] after a 12-year gap to report its new home alongside other important databases for biological classification [20, 21].

The protein section includes updates from two members of the wwPDB consortium [22]. The RCSB paper [23] has a detailed and fascinating report on its incorporation of integrative/hybrid models, i.e. those resulting from multiple experimental and computational methods; while PDBe [24] reports on new clean and stylish entry pages, the result of extensive user feedback, and the welcome facility for users to upload their own residue-level data to view in the context of PDB structures and annotations. The AlphaFold Protein Structure Database also has an update [25] describing a series of significant improvements: alignment to a more recent UniProt [26] release, coverage of isoforms, provision of multiple sequence alignments generated during model construction, and a neat, tabbed interface with integration of results from The Encyclopedia of Domains [27]. Elsewhere, DynaRepo [28] is a new database of molecular dynamics trajectories containing, collectively, over a millisecond of simulation across single proteins and protein complexes. Of course, not all proteins are structured, and the popular DisProt for intrinsically disordered proteins [29] reports how careful extrapolation across species can magnify disorder annotations by two orders of magnitude and illustrates the team’s committed engagement with users, ontologies, and community standards. The proteins underlying phase separation and condensate formation are covered by an updated PhaSepDB [30] that doubles in size as a result of an AI prioritization plus human curation approach, now becoming common; and by CD-CODE [31], appearing in the issue for the first time, that significantly expands to include nucleic acids, condensatopathies, small molecules associated with condensates, and condensates linked to infection. Among resources for domains and families, PROSITE returns [32] after a long hiatus to report better integration with other databases, use of AlphaFold models for domain definition, and some interesting case studies. Elsewhere, the SMART database [33] approaches 30 years with an update illustrating a redesigned user interface and emphasizing the rich context provided by other foundational databases, from STRING [34] to KEGG [35] to Gene Ontology [36]. The update from the popular database of orthology predictions eggNOG [37] describes how the latest version results from a quite different approach that produces groups with greater functional consistency, while the new database, GeneFamily [38], majors on high-quality analysis and visualization tools for diversity, evolution, and genomic context for 2000 mammalian gene families.

In the section for metabolism and signalling, enzymes are a particular focus. The well-established BRENDA reports new features such as gene search and pathway summaries [39], while the new Open Enzyme Database [40] aims to serve as a community repository for enzyme data, offering tools for prediction of kinetic constants and an interesting enzyme recommender based on chemoinformatics analysis of the target substrate. GotEnzymes focuses on predicted enzyme properties, now adding thermal parameters to kinetic constants [41]. Two other new enzyme databases are MITE [42], which offers expert-curated information on the tailoring enzymes that are central to secondary metabolite synthesis; and EnzEngDB [43], which covers enzyme engineering campaigns—users can not only deposit data, but also use helpful tools for visualization and analysis. Two well-established network databases contribute updates: Reactome [44] has a new interface with a range of appealing visualizations, including a clever new disease-versus-normal-state comparison, and an AI chatbot, while SIGNOR [45] showcases a new PhosphoSIGNOR section for phosphorylation-related signalling. They are joined by OmniPath [46], appearing in the issue for the first time, which integrates 168 resources and offers a variety of modes of interactive and programmatic access. Another database arriving in the issue is the foundational MassBank for mass spectral data in metabolomics and environmental chemistry [47]. Elsewhere, QSproteome [48] is a new database for computationally predicted oligomeric protein complexes that offers a user-friendly upload portal, a variety of visualizations and scores, and even a gamified workflow to help engage the community in re-curation efforts. Finally, there is significant expansion reported at FerrDb, a database initially focused specifically on ferroptosis, but which now expands hugely to cover 22 modalities of regulated cell death [49].

The next section covers viruses and microbes, and there are updates from two pillars of microbial taxonomy and systematics. GTDB [50] offers a comprehensive update, including a commitment to extend the current prokaryote-only database to fungi; while another update covers both TYGS and LPSN, which have expanded content, especially for cyanobacteria and medically important bacteria [20]. Another notable update covers the virus taxonomy defined by the ICTV: it details changes to computing infrastructure, improved visualisation tools, and efforts towards training and outreach [51]. Viruses in general are well-represented. For example, two databases cover human viral diseases: an update from ViMIC [52] reports a move from transcriptomics alone to multi-omics and includes two interesting case studies; while the new database scMOVIR [53] offers a single-cell and multi-omics perspective on viral infections and immune responses. Two more new databases focus on metagenome-assembled virus sequences: VIRE [54] links millions of virus genomes and their encoded functions to environments, and benefits from integration with the SPIRE database for metagenome-assembled genomes [55] and the Metalog resource for relevant manually curated metagenome metadata (also debuting in this issue [56]). MetaVR [57] is the successor to IMG/VR [58] and expands its content and diversity significantly, also adding information on eukaryotic hosts as well as AlphaFold-predicted protein structures. Antimicrobial resistance is covered by two new databases: ARKbase [59] is a multi-dimensional database created in response to the publication of the WHO Bacterial Priority Pathogen list, while AMRnet [60] provides comprehensive geographical and temporal visualization of public genomes, their variants and resistance properties. Finally, a number of databases cover the human microbiome. The latest update from GMrepo for the human gut microbiome describes how more abundant data and facilities for comparison between projects allow firmer conclusions on linkages between microbes and phenotypes [61]. Meanwhile, the new gutMSNP [62] focuses on single nucleotide polymorphisms in gut microbes, allowing them to be linked to phenotypes. Finally, another new resource, ResMicroDb [63], comprehensively covers the human respiratory microbiome and provides thousands of microbe-disease associations.

This year, the human, model organism, and comparative genomics section houses both the issue’s Breakthrough articles. The first, JoGo [64], uses long-read sequenced genomes from across the globe to define millions of haplotypes across MANE-standardized human genes [65]. Inspired by conventions for naming HLA alleles, the resource introduces a consistent hierarchical nomenclature, allowing numbering in accordance with global frequency. Links to other databases—ClinVar [66], GWAS Catalogue [67], and GTEx [68]—as well as haplotype-expression QTL results facilitate the identification of variants relevant to gene regulation, disease, and precision medicine. The second Breakthrough article is from So3D [69], a three-dimensional spatial genomics database. Outside a few specialized resources, current databases focus analysis within 2D slices, while So3D carries out analysis between slices in the third dimension. Data include 30 human datasets across 10 organs and multiple diseases, and 33 Drosophila datasets informing on development. A comprehensive range of analytical options includes identification of spatially differentially expressed genes, cell–cell communication, and integration of single-cell datasets to map cell types in 3D. Two other new arrivals, Spatial GWAS Atlas [70] and Spatial2GWAS [71], each link spatial transcriptomics regions to GWAS traits to help elucidate the spatial mechanisms behind complex traits, and thereby facilitate therapeutic target discovery and precision medicine. The 3D chromatin structures ultimately underlying transcriptional regulation are the focus of two databases: enhancer3D [72] interestingly covers ancient as well as modern humans and measures enhancer-promoter distances; while ChromPolymerDB [73] contains single-cell chromatin structures and thereby allows comparisons across cell types, developmental stages, and disease contexts. Elsewhere, two stalwarts of the genomics space, Ensembl and UCSC Genome Browser, each offer their usual annual update. Ensembl [74] reports addition of nearly 2000 new genomes, better treatment of human and barley pangenomes, and improved prediction of the impact of variants; while the Browser [75] has (like Ensembl) absorbed data from MaveDB [76] to guide variant interpretation and has a new tool to facilitate liftover of annotations from one assembly to another. Two major immunogenetics databases also have updates: IPD-IMGT/HLA [77] for HLA system alleles addresses rapid growth with new submission and retrieval tools; and IMGT [78] for immunoglobulins and T-cell receptors reports new presentation, analytical, and predictive tools. There is also an interesting pair of new aquatic databases: iFish [79] ranges widely across 88 species and captures an impressive array of omics data; while Deep Ocean Omics [80] targets the fascinating biology of that extreme environment, with multi-omics data for 68 species across seven phyla. Finally, Genenames.org [81] describes the interesting variety of routes by which official gene names are introduced or altered and showcases a whole new initiative on plant gene names.

In the section on human genomic variation, diseases, and drugs, there are several new interesting cancer databases: CNAScope [82] focuses on copy number aberrations and offers a wide variety of annotations and interactive visualization tools; while PCsRNAdb [83] looks at small ncRNAs in cancer, their (differential) expression, and targets. Elsewhere Rare Cancer Explorer [84] covers 13 unusual tumour types comprehensively, from gene expression to drug response; scCT-DB [85] provides paired scRNA-seq data from patients before and after drug treatment to address perturbation and resistance; and TE-SCALE [86] explores transposable element expression at single-cell resolution across human cancers. They are joined by updates from OncoDB [87], which adds new database modalities such as proteomics and chromatin accessibility, as well as sophisticated tools to explore cross-omics relationships; and, publishing here for the first time, PRECOG [88], which links gene expression to clinical outcome and where there is a new focus on paediatric cancer. Two databases explore human genome variation broadly: the popular FAVOR [89] reports substantial expansion of annotations, a genome browser, and an AI interface to assist the user with navigation and interpretation; while the new IGVF Catalogue [90] assembles diverse experimental and computational data into a vast graph database with three billion nodes. On drug targets, the new database GENEasso [91] helps discover disease-gene associations more reliably through the integration of different statistical models, and the popular Therapeutic Target Database [92] has valuable new data on, for example, the clinical profiles of approved drugs. The effects of protein post-translational modifications on drug sensitivity and resistance are captured by the new PMADS database [93], with thousands of curated links and many more computationally predicted from proteomics data. Drug transporters are covered by VARIDT [94], which now considers their distribution at tissue, cell, and subcellular levels; while mechanisms of drug resistance are dealt with by DRESIS [95], which includes new data on metabolic reprogramming and structural variation, and which contributes our striking cover image. Bridging targets and the drugs themselves is the venerable IUPHAR/BPS Guide to Pharmacology, which, among many improvements, has a new focus on antibacterial pharmacology [96]. Moving on to the drugs themselves, distinct classes of therapeutics gain their own databases: theRNA [97] covers therapeutic RNAs across nine major classes through curation of thousands of papers; while RadioPharm [98] covers the interestingly diverse array of radiopharmaceuticals; and Macrocycle-DB [99] is a specialized resource for the structure, bioactivity, and chemical properties of this important class. Finally, targeted protein degraders of six kinds are covered by TPDdb [100], while no fewer than three new databases capture developments in the specific area of molecular glues [101–103].

The plant database section contains two updates. The first, from Gramene [104], reports valuable new pan-genome subsites for four crops and numerous other improvements; while the Plant Genome Duplication Database [105] reports an eight-fold expansion and hence much better coverage of plant diversity. New databases include GEAR, for diurnal and circadian gene expression in Arabidopsis [106], and two new databases for microbes associated with plants. metagRoot [107] discovers protein families associated with plant root microbiomes, linking them to taxonomy, function, geography, and protein structure; while MicroAgroBiome [108] offers genomes derived from 500 metagenomic studies of the environments of 28 crops. The final section includes updates from two behemoths that defy easy pigeonholing in the previous sections. The Gene Ontology paper describes expansion of several focus areas, such as multi-organism interactions, alongside a new curated set of annotations for human genes, the ‘functionome’ [36]. ChEBI, for curated chemical entities of biological interest, reports a complete reengineering of its web presence in order to allow it to operate in a more automated and sustainable fashion [109]. And bench scientists will appreciate MediaAssist [110], which catalogues the components of serum-free media and co-dependencies, facilitating medium design and optimization.

Finally, a word on changing guidelines around database funding and submission to the database issue. The year has, sadly, seen some acute funding problems strike even major, well-established databases. For the very survival of these crucial resources, such situations may require abrupt shifts to new funding models and hence more restrictive control of access to database content. The database issue has hitherto required that all content of submitting resources be available in some form for free and without the need for users to register. Given the current issues in the sector, we are now introducing some flexibility in exceptional cases. In the future, contributing databases that are forced to charge users and introduce necessary user registration may still be invited to submit to the issue by prior agreement with the editor.

## NAR online molecular biology database collection

As part of our ongoing commitment to maintaining the freely accessible NAR online Molecular Biology Database Collection (http://www.oxfordjournals.org/nar/database/c/), we have continued to monitor and curate the listed resources. During this comprehensive annual review, 899 entries were updated, 96 new resources were added to the database, and 319 outdated or discontinued entries were removed. Additional curation efforts, incorporating recent updates and user feedback, have brought the total number of databases in this release to 2173. We encourage authors to submit their updates to XMF at xose.m.fernandez@gmail.com in plain text, preferably using the template provided at http://www.oxfordjournals.org/nar/database/summary/1.
