# Correction to ‘The 2026 Nucleic Acids Research database issue and the online molecular biology database collection’

**DOI:** 10.1093/nar/gkag439

**Published:** 2026-04-29

This is a correction to: Daniel J Rigden, Xosé M Fernández, The 2026 Nucleic Acids Research database issue and the online molecular biology database collection, *Nucleic Acids Research*, Volume 54, Issue D1, 6 January 2026, Pages D1–D9, https://doi.org/10.1093/nar/gkaf1427

The following change has been made to the originally published paper.

In Table 1 of this NAR database summary paper, the URL link for Data Race column line “RaCE” was incorrect and has been corrected to read: https://biospace.shinyapps.io/race/.

